# Checkpoint kinase inhibitor AZD7762 enhance cisplatin-induced apoptosis in osteosarcoma cells

**DOI:** 10.1186/s12935-019-0896-9

**Published:** 2019-07-27

**Authors:** Jian Zhu, Hanhui Zou, Wei Yu, Yuluan Huang, Bing Liu, Tao Li, Chengzhen Liang, Huimin Tao

**Affiliations:** 1 Department of Orthopedics, 2nd Affiliated Hospital, School of Medicine, Zhejiang University, #88 Jie Fang Road, Hangzhou, 310009 Zhejiang People’s Republic of China; 2 Orthopedics Research Institute of Zhejiang University, #88, Jiefang Road, Hangzhou, 310009 China; 3 Department of Gynecologic Oncology, Women’s Hospital, School of Medicine, Zhejiang University, Hangzhou, China; 4 Dept Bone & Soft Tissue Surg, Zhejiang Canc Hosp, 38 Guangji Rd, Hangzhou, 310022 Zhejiang People’s Republic of China

**Keywords:** AZD7762, Cisplatin, Osteosarcoma, Apoptosis, Chk1

## Abstract

**Background:**

AZD7762 is a checkpoint kinase 1 (Chk 1) inhibitor, which has been reported to sensitize many tumor cells to DNA damage. However, whether AZD7762 could sensitize osteosarcoma cells to chemotherapy cisplatin has not been defined.

**Methods:**

We used a variety of methods such as cell viability assays, flow cytometry, western blotting, and immunohistochemistry analysis to determine AZD7762 enhancing cisplatin-induced apoptosis on osteosarcoma cell lines in vitro and in vivo.

**Results:**

In the present study, we demonstrated that AZD7762 could enhance cisplatin-mediated apoptosis and mitotic catastrophe of osteosarcoma cells in vitro, and promote the inhibition of xenograft growth induced by cisplatin in vivo. The mechanistic study indicated that AZD7762 enhance the effect of cisplatin through abrogating cisplatin-mediated G2/M arrest and inhibiting the cisplatin damage repair as demonstrated by increasing cisplatin-induced γH2AX expression.

**Conclusion:**

These results suggest that AZD7762 could effectively promote cisplatin-induced apoptosis and mitotic catastrophe in osteosarcoma cells. The clinical application of AZD7762 as an adjuvant in the chemotherapy of osteosarcoma should be further explored.

**Electronic supplementary material:**

The online version of this article (10.1186/s12935-019-0896-9) contains supplementary material, which is available to authorized users.

## Background

Osteosarcoma (OS) is the most common primary malignant tumor of bone, occurring predominantly in children and adolescents with a very high propensity for local invasion and early systemic metastases. The highest-risk population is children aged 5 to 15, and it has become the third cause of cancer-related death in adolescents [1]. With current therapies of OS, such as extensive surgical excision, radiotherapy, and neoadjuvant chemotherapy, the 5-year survival rate has increased up to 50–60%. By contrast, in the metastatic and recurrent conditions, the long-term survival rate of osteosarcoma patients still remains at 10–20% [2]. At the same time, the high-dose use of chemotherapeutic drugs is limited because of their systemic toxicity and drug resistance, which become major obstacles for the treatment of osteosarcoma [3, 4]. Therefore, the development of novel therapies for the management of osteosarcoma is especially urgent.

Cisplatin is a commonly used adjuvant chemotherapeutic drug for osteosarcoma treatment. Cisplatin could bind to purine and pyrimidine bases of DNA to form crosslinks between chains, which damages cellular DNA and causes cancer cells to die. The cytotoxicity of cisplatin involves the formation of intra and interstrand DNA adducts, and the resulting DNA damage triggers apoptosis. However, DNA damage also activates cell cycle proteins, arresting the impaired cell cycle and allowing time for DNA repair [5, 6]. The damage of DNA can activate multiple cell cycle checkpoints (G1/S, S, G2/M) and arrest the cell cycle at the detection point for DNA repair. This is a cell’s self-protection mechanism, and inhibition of this mechanism can enhance the role of the chemotherapeutic drug.

Checkpoint protein kinases (Chk) Chk1 and Chk2 play a key role in regulating cell cycle. The activation of Chk1 is responsible for S phase and G2/M phase block, while Chk2 is generally considered to play a role in detection effect amplification [7, 8]. Unlike Chk2, Chk1 plays a key role in maintaining DNA integrity. During cell cycle arrest induced by DNA damage, activation of Chk1 could induce the phosphorylation of Cdc25 phosphatase family, which inhibits the activation of the regulatory protein Cdc2. Therefore, the tumor cells can be arrested at the cycle detection point until the damaged DNA is repaired.

Recently, a novel selective Chk1 and Chk2 inhibitor 1-(2-((*S*)-piperidin-3-ylcarbamoyl)-5-(3-fluorophenyl) thiophen-3-yl)urea (AZD7762) has entered clinical tests [9, 10]. Chk1 inhibitors AZD7762 significantly potentiated the cytotoxic effects of gemcitabine, cisplatin and paclitaxel [8]. AZD7762 could enhance the effect of cisplatin on tumor cells, including breast cancer [11], ovarian cancer [12, 13], head and neck cancer [14] and medulloblastoma [15]. However, the role of Chk inhibitors AZD7762 in combination with cisplatin in osteosarcoma has not been studied. Therefore, this study aims to verify whether AZD7762 can enhance the chemotherapy effect of cisplatin in osteosarcoma, and to explore the underlying mechanism.

## Materials and methods

### Regents and antibodies

AZD7762 was from Selleck Chemicals (Houston, TX, USA). The chemical formula of AZD7762 is C17H19FN4O2S. Cisplatin was purchased from SigmaAldrich (St. Louis, MO, USA). AZD7762 and cisplatin were separately dissolved in PBS to produce a stock solution. The AZD7762 and cisplatin stock solution were diluted in a cell culture medium prior to its use in each experiment. Trypsin (0.25%), PBS, fetal bovine serum (FBS), Eagle’s Minimum Essential medium (EMEM) and Mcroy’ 5A medium were from Gibco; Thermo Fisher Scientific, Inc. (Waltham, MA, USA). MTS kit was obtained from Promega Corporation (Madison, WI, USA). Antibodies against cleaved-caspase-3 (Cat. no. 9661), cleaved-caspase-9 (Cat. no. 9505), poly (ADPribose) polymerase (PARP; Cat. no. 9532), cyclin B1 (Cat. no. 4135), Bcl-2 (Cat. no. 4223), Bcl-2 X-associated protein (Bax; Cat. no. 5023), phosphorylated-Chk1 (Cat. no. 2348), phosphorylated-Cdc25C (Cat. no. 4901), Phospho-Histone H2A.X (Cat. no. 9718), phosphorylated-Cdc2 (Cat. no. 4901) and GAPDH (Cat. no. 5174) were from Cell Signaling Technology, Inc., (Beverly, MA, USA).

### Cell and cell culture

The human osteosarcoma cell lines MNNG/HOS (CRL-1547TM, ATCC), Saos-2 (HTB-85TM, ATCC) were obtained from Cell Bank of Shanghai Institute of Biochemistry and Cell Biology, Chinese Academy of Sciences (Shanghai, China). Saos-2 cells were cultured in McCoy’5A medium (Invitrogen GIBCOLTM) containing 10% fetal bovine serum and 100 U/mL of penicillin/streptomycin. HOS cells were incubated in EMEM containing 10% fetal bovine serum and 100 U/mL penicillin/streptomycin (Invitrogen GIBCOL TM). Cells were inoculated into 25 cm^2^ cell culture flasks and cultured in a 37 °C incubator containing 5% CO_2_. Both the control group and drug-treated group cells were treated with serum-containing media.

### MTS assay for inhibition of cell proliferation

The cells in logarithmic growth phase were washed with sterile PBS and digested with trypsin containing EDTA. The osteosarcoma cells were seeded in a 96-well plate with a density of 4–5 × 10^3^ cells/well and cultured for 24 h to adhere cells. After discarding the culture medium and washing with PBS, 100 μL of culture medium containing the different concentration of cisplatin (1.25 μmol/L to 80 μmol/L) was added to each well of the test group and the control group for 24 h. After the medium was discarded and washed with PBS, 100 μL medium containing 100 nmol/L AZD7762 was added to each well of the test group and cultured for 24 h. Then MTS solution was added and incubated in an incubator containing 5% CO_2_ and 37 °C for 2–3 h. The absorbance OD was measured at a wavelength of 490 nm using an enzyme-linked reaction detector. The difference of inhibition rates between the test group and the control group was compared. The median inhibitory concentration (IC50) was calculated using the Logit method. The data was averaged in six replicates, and each experiment repeated for three times.

$$ \begin{aligned}&  {\text{Cell survival rate }} \\ &\quad = \, \left( {{\text{OD treatment group }} - {\text{ OD blank group}}} \right)/\\ &\qquad\left( {{\text{OD control group }} - {\text{ OD blank group}}} \right). \end{aligned} $$

### Flow cytometry cell cycle assay

Osteosarcoma cells were seeded at a density of 2 × 10^4^ cells/well in a 6-cm dish and cultured for 24 h to adhere cells. In the experimental group and the control group, 2 mL of the culture medium containing cisplatin (10 μmol/L) was added, and the incubator was cultured for 24 h. Then the culture medium was removed and washed twice with PBS. The test group was supplemented with 2 mL of medium containing 100 nmol/L AZD7762, and the control group was supplemented with 2 mL of medium for 24 h. Then cells were fixed in − 20 °C overnight with 75% ethanol. The fixed cells were incubated with the PI reagent for 15 min at room temperature in the dark and tested on the flow cytometry (FACSCalibur; BD Biosciences, San Jose, CA, USA). The results were analyzed by ModFit software (version 3.3; Verity Software House, Topshame, ME, USA). The experiment was repeated for three times.

### Western blot experiment

Cells were cultured in 6-well plates at a density of 3 × 10^5^ cells/well. Following seeding, cells were treated with 100 nmol/L AZD7762, 10 μmol/L cisplatin, or a combination of both for 24 h. After 24 h, the culture medium was removed and washed twice with PBS. Cells were lysed on ice with RIPA lysate containing PMSF (protease inhibitor) and the supernatant was collected after lysis on ice for 30 min. The concentration of protein was quantified using the BCA Protein Concentration Detection Kit. Total proteins (40 µg) protein was separated by 8–12% SDS-PAGE at 80 V for 1–1.5 h. Protein was transferred to 2.2 µm PVDF membranes at 250 mA for 2 h. The PVDF membranes were blocked with bovine serum albumin for 30 min and incubated with primary antibody (CST antibody dilution ratio 1:1000) in a gentle horizontal shaker overnight. After rinsing five times with TBST solution for 5 min each time, the PVDF membranes were incubated with the secondary antibody (1:5000 dilution) in a gentle shaker for 1.5 h at room temperature. The bands were developed by enhanced chemiluminescence kit (Millipore).

### Xenograft orthotopic model

Male BALB/c-nu mice (16–18 g) were purchased from Shanghai Laboratory Animal Center of Chinese Academy of Sciences. Mice were maintained under specific pathogen-free conditions with sterilized food and water. HOS cells were transfected with luciferase (HOS-luc) for in vivo imaging. Each mouse was injected with HOS-luc cells (5 × 10^6^) in the right tibia. An in vivo bioluminescence imaging system was used to measure luminescence intensity. When the luminescence intensity reached 1 × 10^6^ p/s, mice were randomly divided into four groups (five mice per group). Mice with a very low or high luminescence signal were killed. Then, AZD7762 was dosed intraperitoneally daily at 25 mg/kg and cisplatin was dosed at 6 mg/kg intraperitoneally every 3 days for the treatment. The luminescence intensity was measured every 5 days. After 15 days of dosing, all mice were killed. The tumors were excised and fixed in 4% paraformaldehyde for further analysis. All treatments were approved by the Research Ethics Committee of the Second Affiliated Hospital of Zhejiang University School of Medicine, China.

### In vivo bioluminescence assay

200 μL of luciferin (15 mg/mL) was injected intraperitoneally into the mice 10 min before performing in vivo imaging according to the manufacturer’s instructions. Mice were anaesthetized with isoflurane. An IVIS 200 imaging system was used to perform in vivo imaging, and the results were analyzed with Living Image Software (Version 3.0.4, Xenogen, Hopkinton, MA, USA). The total flux of the region of interest was measured as photons (p)/s for each mouse.

### Tumor histology

The fixed tumor tissue was dehydrated with gradient increased alcohol and embedded in paraffin. Tumor tissue was cut into serial sections (3 μm) and deparaffinized. Then slides were stained with hematoxylin and eosin.

### Immunohistochemistry analysis

Sections were deparaffinized and incubated with 3% H_2_O_2_ for 15 min to block endogenous peroxidase activity. Next, sections were immersed in boiling sodium citrate buffer (pH 6.0) for antigen retrieval. Then, the slides were blocked with 5% BSA for 30 min at 37 °C and incubated with first antibodies against cleaved caspase 3, cleaved caspase 9, Bax, Ki-67, p-Chk1, p-Cdc25C, p-Histone H2A.X at 4 °C overnight. Then the slides were incubated with biotin-labelled secondary antibody at 37 °C for 30 min. Immunoreactivity was detected by SABC method. The results were recorded by a DP70 CCD camera (Olympus) and an AX-70 microscope (Olympus). Digital images were analyzed by image-pro-plus (version. 6.0, Media Cybernetics). The measure parameters contained mean density, total area and IOD. The optical density was calibrated and the area of interest assigned value for hue, 0–30; saturation, 0–255; intensity, 0–230. Then the image was transformed into a grey-scale image to measure the values.

### Statistical analysis

The data were expressed as mean ± SD. The data differences were analyzed with one-way ANOVA and Student’s t-test. Data analysis was performed in SPSS software (version 19.0; SPSS, Inc., Chicago, IL, USA). p < 0.05 indicates a significant difference.

## Results

### The inhibitory effect of AZD7762 on the proliferation of human osteosarcoma cell lines

**Fig. 1:**
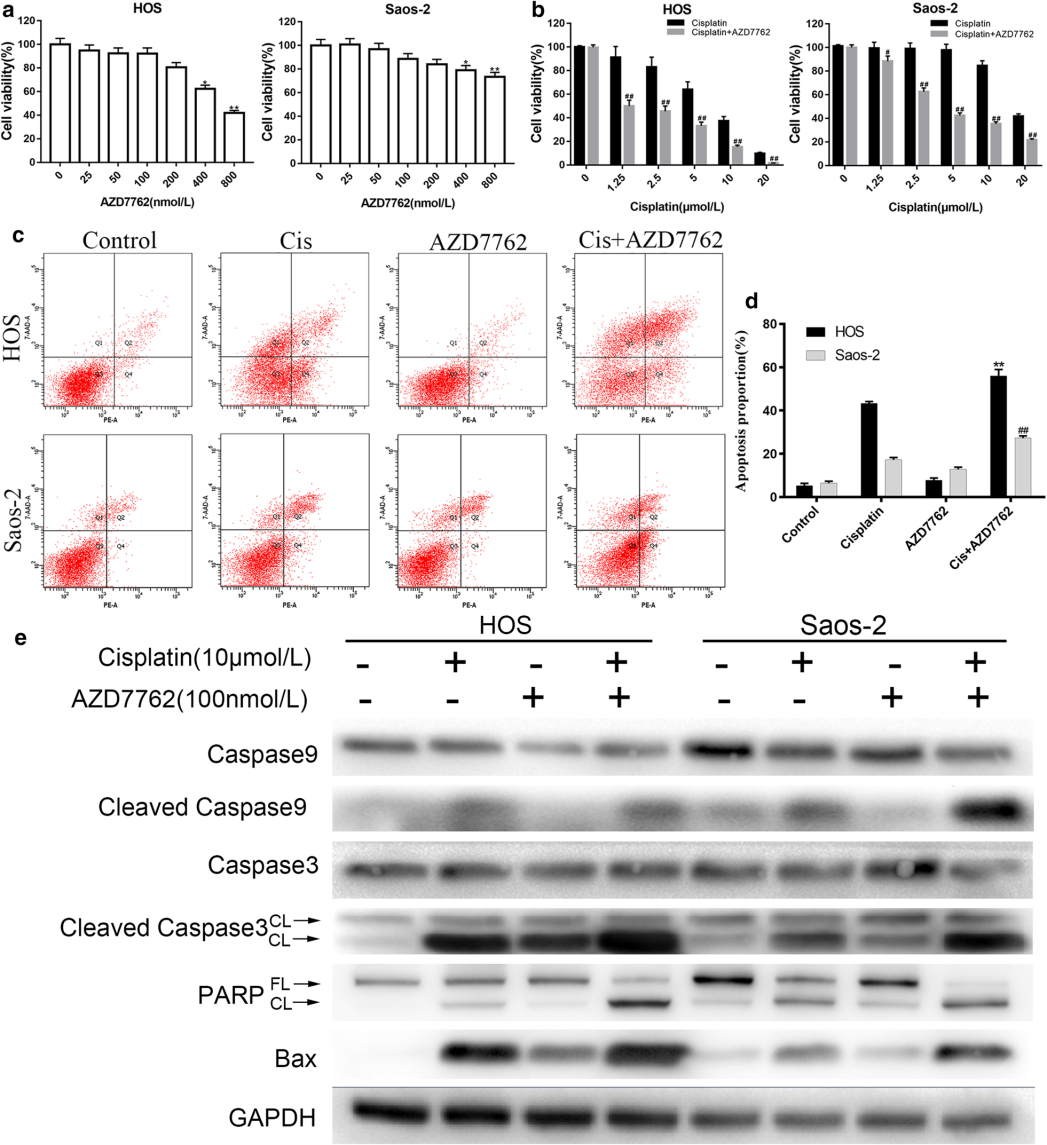
AZD7762 enhance the effect of cisplatin on human osteosarcoma cells. **a**, **b** MTS assay analyzes the inhibitory effect of AZD7762 and cisplatin on the proliferation of osteosarcoma cell lines. HOS and Saos-2 cell were treated with different concentration of AZD7762 and cisplatin for 24 h, respectively. The results are the mean of six replicates, and each experiment performed in triplicate. **c, d** The inhibitory effect detected by MTS assay that AZD77762 (100 nmol/L) in combination with different concentration of cisplatin on HOS and Saos-2 cells for 24 h. The results are the mean of six replicates, and each experiment performed in triplicate. **e** Human osteosarcoma cells HOS and Saos-2 were treated with PBS (control group), cisplatin (10 μmol/L), AZD7762 (100 nmol/L) and cisplatin + AZD7762 for 24 h. The expression level of apoptosis-related protein PARP, cleaved caspase-3, -9 and Bax were determined by western blot. *p < 0.05 and **p < 0.01 versus control group. ^#^p < 0.05 and ^##^p < 0.01 versus cisplatin treatment with the same concentration. *FL* full length, *CL* cleaved

The inhibitory effect of Chk1 inhibitor AZD7762 was studied on the proliferation of two human osteosarcoma cell lines. As shown in Fig. 1a, MTS assay showed that AZD7762 had no significant inhibitory effect on the proliferation of human osteosarcoma cell lines at low concentrations (≤ 200 nmol/L), and AZD7762 inhibited the two cell lines at higher concentrations (> 200 nmol/L). The effect appeared dose-dependent. The IC50 of AZD7762 on HOS and Saos-2 cell lines were 550 nmol/L and 2.3 μmol/L on 24 h, respectively. For further experiments, we determined the concentration of AZD7762 to be 100 nmol/L based on the results of MTS and according to the referenced literature. At this concentration, AZD7762 had no significant inhibitory effect on the two osteosarcoma cell lines.

### AZD7762 combined with cisplatin significantly inhibits proliferation of osteosarcoma cell lines

The inhibitory effect of AZD7762 (100 nmol/L) combined with cisplatin on two human osteosarcoma cell lines was detected by MTS. The IC50 of cisplatin alone in HOS and Saos-2 cells was 8.82 μmol/L and 20.64 μmol/L, respectively (Fig. 1b). Compared with cisplatin alone, AZD7762 (100 nmol/L) combined with cisplatin significantly enhanced the inhibitory effect of cisplatin on two human osteosarcoma cell lines. The IC50 of cisplatin and AZD7762 in HOS and Saos-2 cells was 3.28 μmol/L and 8.04 μmol/L, respectively (Fig. 1c). This enhanced inhibition was also evident at very low cisplatin concentrations and significantly increased the sensitivity of HOS and Saos-2 osteosarcoma cells to cisplatin.

### AZD7762 enhances apoptosis of osteosarcoma induced by cisplatin

Annexin-PE/PI staining results indicated that cisplatin combined with AZD7762 induced more apoptosis of osteosarcoma cell lines than cisplatin alone (Fig. 1b, c). As showed in Fig. 1e, AZD7762 significantly increased the expression of apoptosis-related proteins in tumor cells. In apoptosis, caspase is the principal enzyme that performs apoptosis. As previously reported, caspase-9 is usually considered to be the starting factors of apoptosis, and caspase-3 acts as the activation factor of apoptosis which cleaves PARP [16]. Compared with the control group, cisplatin significantly increased the expression of caspase-3 and caspase-9 in HOS and Saos-2 cells and up-regulated the cleavage and expression of its substrate PARP. The combination of AZD7762 increased the expression of caspase-3, -9 and PARP induced by cisplatin. At the same time, we also detected that AZD7762 also increased the expression of cisplatin-mediated mitochondrial apoptosis-related protein Bax. Furthermore, AZD7762 decreased the expression of Bcl-2 and increased the expression pf Bak mediated by cisplatin (Additional file 1).

### AZD7762 reduces cisplatin-mediated G2/M cell cycle arrest

**Fig. 2:**
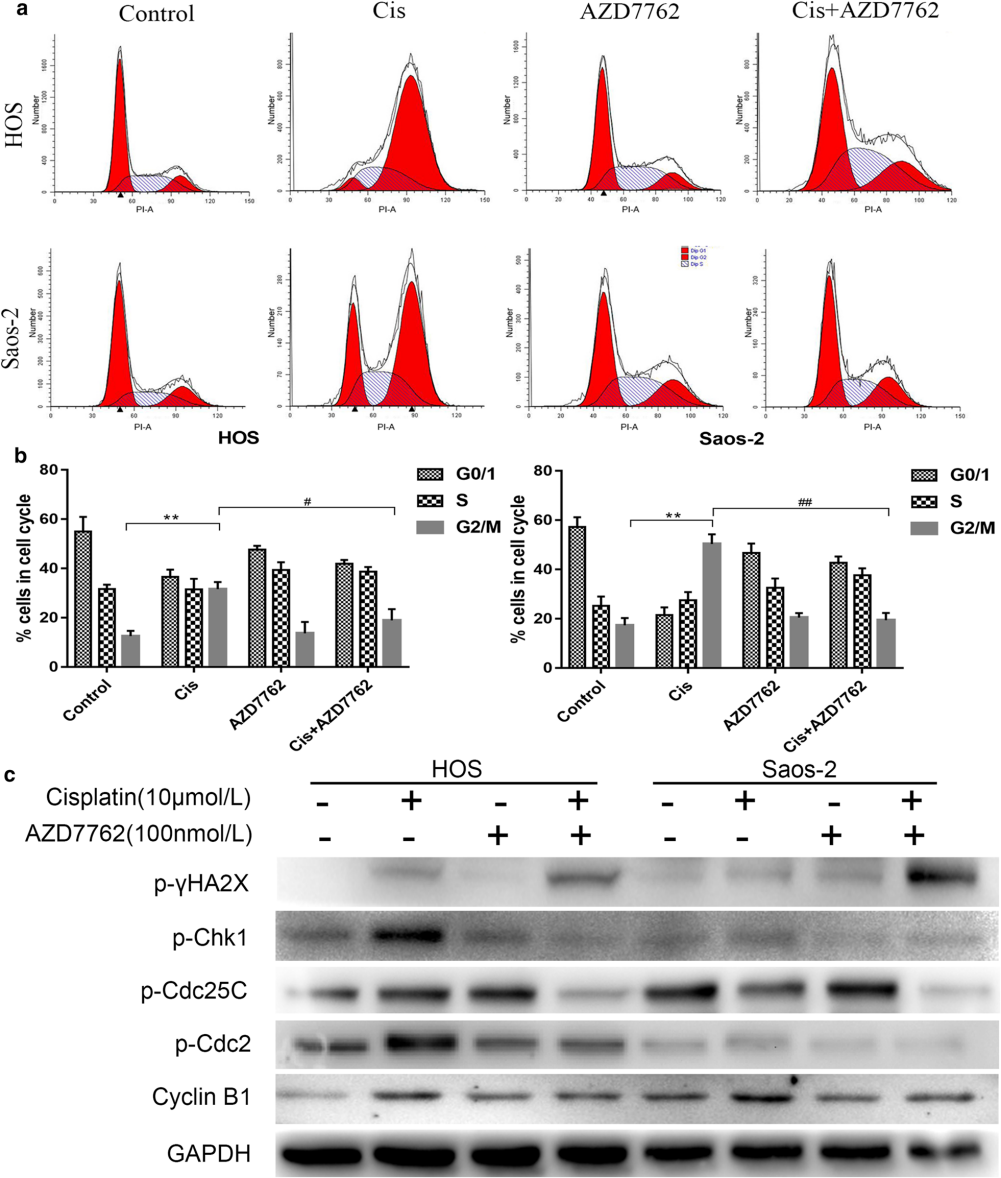
Analysis of AZD7762 in combination with Cisplatin on cell cycle of osteosarcoma cells. **a**, **b** HOS and Saos-2 cells were treated with PBS (control group), cisplatin (10 μmol/L), AZD7762 (100 nmol/L) and cisplatin + AZD7762 for 24 h. The data of cell cycle distribution detected by flow cytometry were obtained from three independent experiments. *p < 0.05 and **p < 0.01 versus control group. **c** Human osteosarcoma cells HOS and Saos-2 were treated with cisplatin (10 μmol/L) alone or in combination with AZD7762 (100 nmol/L) for 24 h. The expression of cell cycle related protein of p-Chk1, p-Cdc25C, p-Cdc2, Cyclin B1 and phosphorylated histone H2A.X were measured by western blot. ^#^p < 0.05 and ^##^p < 0.01 versus cisplatin treatment with the same concentration

AZD7762 is an inhibitor of Chk1 which regulates the cell cycle. Therefore, we explored whether AZD7762 enhances the inhibition effect of cisplatin on osteosarcoma proliferation through regulating the cell cycle. HOS and Saos-2 cells were treated with PBS (control group), cisplatin (10 μmol/L), AZD7762 (100 nmol/L), and cisplatin + AZD7762 for 24 h, and flow cytometric analysis was performed (Fig. 2a). Compared with the control group, the G2/M phase of HOS and Saos-2 cells treated with cisplatin was significantly increased. When AZD7762 was combined with cisplatin, the number of cells in the G2/M phase was significantly reduced (Fig. 2b). Although there was a decrease in the G1/0 phase and an increase in the S phase ratio in both cells treated with AZD7762 alone, there was no significant difference.

### AZD7762 combined with cisplatin down-regulates cell cycle checkpoint protein expression

To investigate the mechanism that AZD7762 reducing cisplatin-mediated G2/M cell cycle arrest in the human osteosarcoma cell lines HOS and Saos-2 cells, the protein of p-Chk1, p-Cdc25c, p-Cdc2, p-histone H2A.X and Cyclin-B1 that involved in G2/M cell cycle regulation were performed in western blot experiments. Cisplatin significantly increased the phosphorylation of protein kinase Chk1, Cdc25c, Cdc2 and histone H2A.X in HOS and Saos-2 cells and upregulate the expression level of Cyclin-B1 compared to the control group (Fig. 2c). On the contrary, when both cells were treated with cisplatin combined with AZD7762, the expression levels of p-Chk1, p-Cdc25c, p-Cdc2 and Cyclin B were significantly decreased and p-histone H2A.X was upregulated. The relative expression of P-Cdc2, P-Chk1 and cyclin B1 in saos-2 cells were shown in Additional file 2. This result indicated that AZD7762 reduces arrest of G2/M phase mediated by cisplatin through regulating the cell cycle checkpoint protein expression.

### Cisplatin combined with AZD7762 reduced tumor growth in vivo

**Fig. 3:**
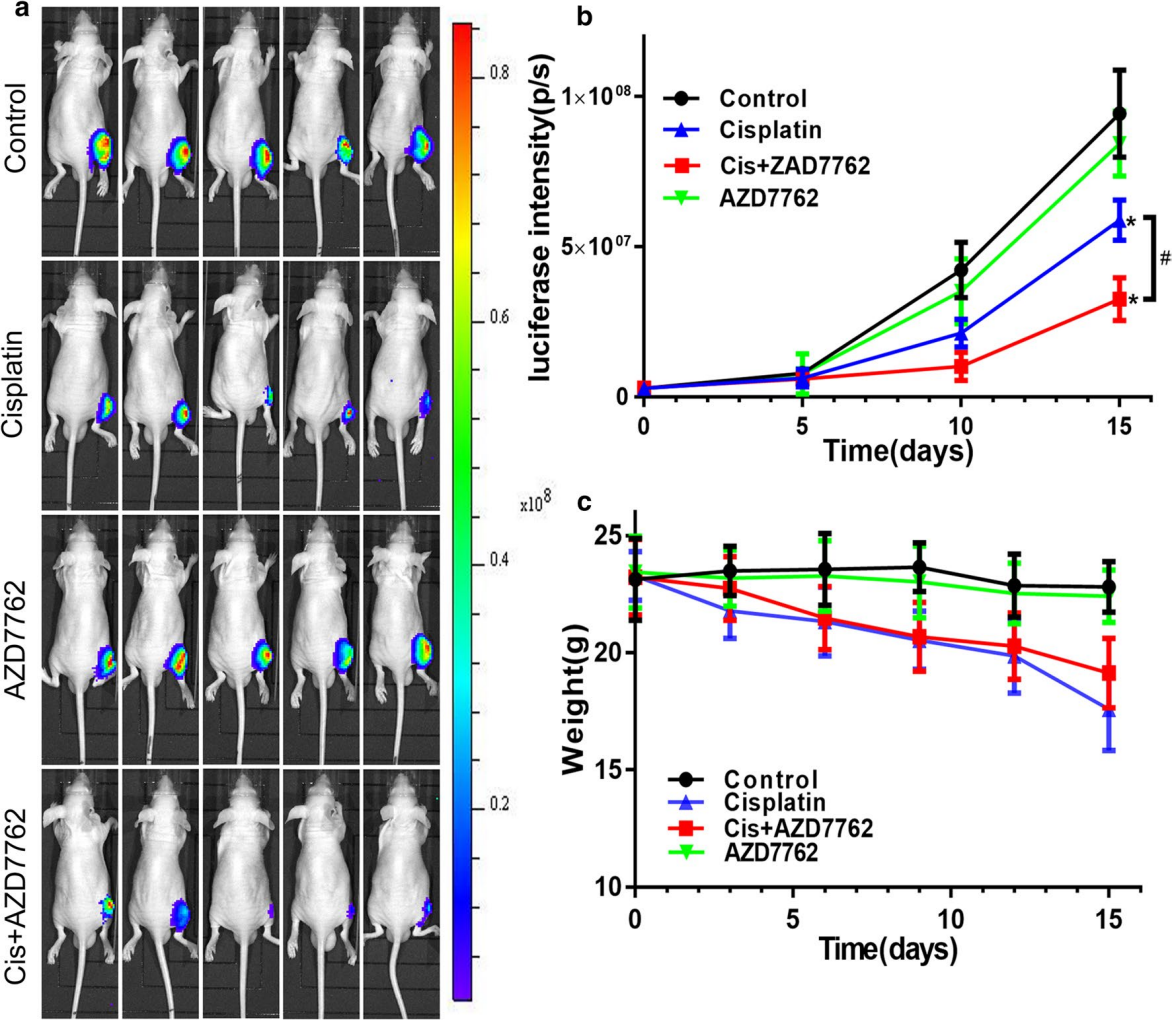
Cisplatin combined with AZD7762 inhibits the growth of xenografts in vivo. **a**, **b** The luminescence intensity was used as an indicator of tumour size by in vivo imaging system. And luciferase intensity was calculated using the in vivo imaging software. **c** Body weight was measured every 3 days

**Fig. 4:**
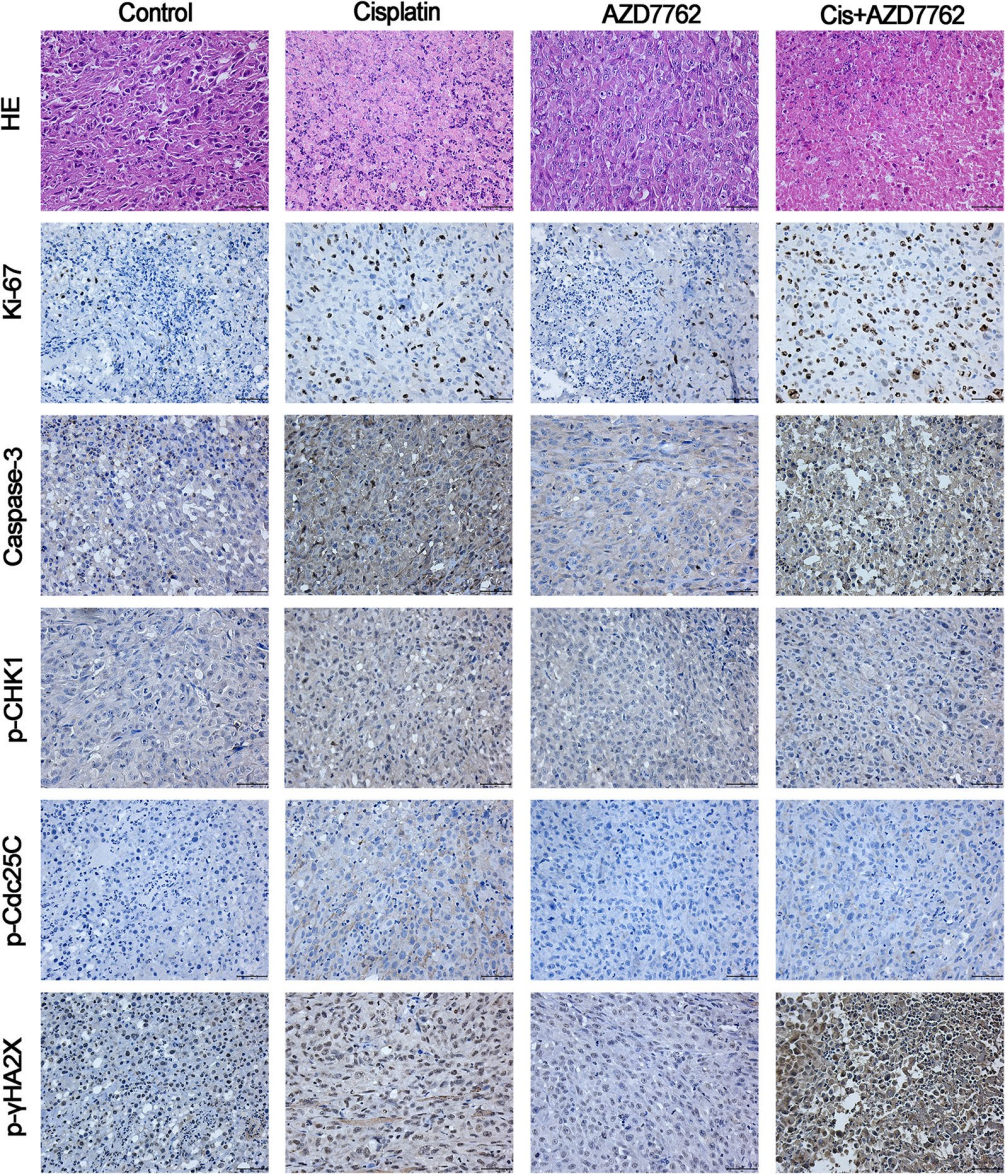
Histology and apoptotic status of tumor tissues were evaluated with H&E staining, Ki-67 expression. Immunohistochemistry was used to assess the levels of cleaved caspase-3, p-Chk1, p-Cdc25C, and p-histone H2A.X. Representative images are presented. Scale bar, 50 μm

In order to simulate the circumstance of osteosarcoma in vivo, an orthotopic model was established through inoculating the osteosarcoma cell into the tibia of nude mice. HOS cells were transferred with luciferase so that tumor size could be calculated by the luciferin intensity. The tumor luminescence intensity showed a significant difference between cisplatin and cisplatin combined with AZD7762 (Fig. 3). The H&E and Ki-67 staining showed more dead cells and apoptosis proportion in cisplatin combined with AZD7762 treated tumor tissues compared to cisplatin used alone. Cisplatin combined with AZD7762 decrease the expression of p-Chk1 and p-Cdc25c and upregulate the expression of cleaved caspase-3, and p-histone H2A.X compared to cisplatin, the immunohistochemical analysis results were consistent with the western blot results (Fig. 4). The high-resolution immunohistochemistry images of p-γHA2X were shown in Additional file 3. These results indicated that AZD7762 enhances the effect of cisplatin in inhibiting the growth of osteosarcoma in vivo.

## Discussion

The cell cycle checkpoint kinase Chk1/2 plays a crucial role in cell cycle regulation. After DNA damage occurs, Chk1 and Chk2 are phosphorylated by ATM and ATR, causing cell cycle arrest and activating DNA damage repair system to maintain cell genome stability [17]. Although this DNA damage regulatory system plays a key role in maintaining genetic stability in normally proliferating cells, it can also be used by tumor cells to evade death [18]. A major feature of cell malignancy is dysregulation of the cell cycle. As the previous study reported, Saos-2 is p53 deficiency, and HOS cell is p53-mutation [19]. Non-functional p53 was expressed in HOS and Saos-2 cells [20, 21]. Deletion of the P53 gene often occurs in tumor cells [22] and results in the loss of G1-S cell cycle detection sites, making tumor cells particularly dependent on G2-M phase detection sites to perform DNA repair [23]. Therefore, the dysregulated expression of Chk1/2 plays an important role in the proliferation of tumor cells and resistance to genotoxic drugs. In particular, some studies found that the expression of Chk1 was often low in normal cells but was high in certain malignant tumors [24]. Our study is based on the above background, inhibiting the activity of cell cycle detection point kinase Chk1 can increase the sensitivity of tumor cells to DNA toxic drugs, and enhance the effect of chemotherapeutic drugs on tumor cells.

AZD7762 is a novel ATP-competitive cell cycle detection point kinase inhibitor, which has a strong inhibiting effect on Chk1. AZD7762 could inhibit phosphorylation of Cdc25C peptide by reversible binding to the ATP binding site of Chk1 [25]. Moreover, AZD7762 is 100 times more selective binding to Chk1 than the traditional Chk inhibitor UCN-01. Several studies have confirmed that AZD7762 can abolish DNA damage-mediated cell cycle detection in many cell lines, including breast cancer, ovarian cancer, urothelial carcinoma, head and neck cancer, medulloblastoma and lung adenocarcinoma. AZD7762 acts in synergy with cisplatin in reducing cell proliferation in medulloblastoma [15]. AZD7762 in combination chemotherapy represent a promising option for the treatment of triple-negative breast cancer [11]. AZD7762 enhanced sensitivity of urothelial carcinoma cells to gemcitabine by inhibiting DNA repair [26]. AZD7762 sensitizes p53 deficient squamous cell carcinoma of the head and neck to cisplatin through induction of mitotic cell death [14]. And AZD7762 could enhance the effect of chemotherapy/radiotherapy on tumor cells and overcome radiotherapy/chemotherapy resistance of tumor cell [8, 11, 27–33]. However, the role of AZD7762 combined with DNA damage drugs in osteosarcoma has not been studied. Therefore, we choose clinical osteosarcoma patients commonly used drug cisplatin for the following studies. The effect and mechanism of Chk1 inhibitor AZD77762 combined with chemotherapeutic drugs in osteosarcoma cell lines were verified through comparing single drug and combined effect. The results may provide new ideas and protocols for clinical treatment of bone tumors.

We first studied the proliferation inhibitory effects of Chk1 inhibitor AZD7762 alone on HOS and Saos-2 cells. We found that AZD7762 had no significant inhibitory effect on the proliferation of human osteosarcoma cell lines at low concentrations (≤ 200 nmol/L). We then verified that AZD7762 combined with cisplatin enhances the inhibitory effect of cisplatin on osteosarcoma cell proliferation. We have found that AZD7762 can significantly enhance the cytotoxicity of cisplatin on both osteosarcoma cells compared to cisplatin alone. Combination therapy significantly increased the IC50 of cisplatin (p < 0.05). In vivo study, tumor size calculated by the luciferin intensity showed a significant difference between cisplatin and cisplatin combined with AZD7762. However, tumor position is very essential for IVIS signal, tumor volume data will be most accurate if tumors are dissectible. In future work, we should further calculate the tumor volume when the tumor is dissectible. These results indicated that AZD7762 may reduce the amount of cisplatin in clinical work, accordingly reducing the incidence of cisplatin dose-dependent adverse reactions.

Cisplatin could induce apoptosis of osteosarcoma cells via caspase-mediated apoptosis pathways [34]. So we use the western bolt to test whether AZD7762 could increase the incidence of cisplatin-mediated apoptosis to enhance the effect of cisplatin. Compared with cisplatin alone, combined AZD7762 significantly increased caspase-3, -9 activation and PARP shear in both cell lines. We also found that the combination of AZD7762 and cisplatin significantly increased the expression of the mitochondrial apoptotic protein Bax. In previous studies, Bak/Bax clusters serve as a channel-like pore on mitochondrial outer membrane and enable small molecules such as cytochrome C to enter the cytoplasm to trigger and activate the intrinsic apoptosis pathway [35, 36]. Autophagy is an evolutionally conserved catabolic process which is necessary for cancer cells to adapt to an unfavorable tumor microenvironment. Accumulating evidence suggests that autophagy has a close relationship with programmed cell death [37]. However, we did not conduct in-depth research on this issue. In future work, we can further explore this mechanism and pathway.

We further explore the effect of AZD7762 and cisplatin on the cell cycle of HOS and Saos-2 osteosarcoma cells by flow cytometric analysis. As expected, we found that cisplatin caused numerous G2/M cell cycle arrest, whereas AZD7762 significantly abolished the G2/M phase cell cycle arrest mediated by Chk1 activation. We also found that the use of AZD7762 alone can increase the proportion of cells in the S phase, although there was no significant difference at a concentration of 100 nmol/L AZD7762, this phenomenon became more and more obvious as the concentration of AZD7762 increased (data not shown).

Many studies have shown that Chk1 mainly plays a role in the G2/M phase detecting point. The regulation of the G2/M phase detecting point by Chk1 was mainly achieved through the ATR-Chk1-Cdc25C-Cdc2(Cdk1)/Cyclin-B1 pathway [24], and we confirmed that AZD7762 could regulate the key protein of this pathway. Compared with the control group, cisplatin significantly increased the phosphorylation of Chk1, Cdc25C and Cdc2 in HOS and Saos-2 cells, while up-regulated the expression Cyclin-B1, causing cell G2/M arrest. When cisplatin was combined with the Chk1 inhibitor AZD7762, we detected a significant decrease in p-Chk1, p-Cdc25C, p-Cdc2, and Cyclin-B1 expression, suggesting that AZD7762 inhibits G2/M cell cycle detection sites, which reduces cell cycle arrest. We also noted that AZD7762 significantly increased cisplatin-mediated phosphorylation of histone H2A.X (p-histone H2A.X). Histone H2A.X is a histone variant that accounts for only 10% of histone H2A in normal human fibroblasts. Histone H2A.X plays an important role in the cell cycle arrest mediated by cell cycle monitoring and repair after DNA double-strand breaks [38]. In addition to its role in DNA damage repair, H2A.X also plays a key role in DNA fragmentation during apoptosis and can be phosphorylated by a variety of apoptosis-related kinases [39–41]. Therefore, the up-regulation of p-Histone H2A.X indicates that there is a large amount of DNA damage and the occurrence of apoptosis-related pathways. The above results indicate that the Chk1 inhibitor AZD7762 enhances cisplatin-mediated cytotoxicity and inhibits the onset of G2/M phase cell cycle arrest caused by DNA damage. Interestingly, AZD7762 alone did not show changes in the expression of other proteins in the Chk1-Cdc25C-Cdc2(Cdk1)/Cyclin-B1 pathway in both osteosarcoma cell lines, which may be due to no occurrence of a large number of cell cycle arrests.

## Conclusion

In summary, our study confirmed that AZD7762 can reduce DNA damage induced by cisplatin through inhibiting the activation of G2/M cell cycle checkpoints and regulating Chk1-Cdc25C-Cdc2/Cyclin-B1 cell signaling pathways. The G2/M phase cell cycle arrest enhances the cytotoxicity of cisplatin on osteosarcoma cells and increases the occurrence of cisplatin-mediated apoptosis. The Chk1 inhibitor AZD7762 is expected to be combined with standard DNA damage chemotherapy drugs as a new option for the treatment of osteosarcoma.

## Additional files

**Additional file 1.** Human osteosarcoma cells HOS and Saos-2 were treated with PBS (control group), cisplatin (10 μmol/L), AZD7762 (100 nmol/L) and cisplatin + AZD7762 for 24 h The expression level of protein Bcl-2 and Bak were determined by western blot.

**Additional file 2.** The relative expression of P-Cdc2, P-Chk1 and cyclin B1 in saos-2 cells were shown with bar graph. *p < 0.05 versus cisplatin treatment with the same concentration.

**Additional file 3.** The high-resolution Immunohistochemistry images of p-γHA2X.

## Data Availability

The datasets supporting the conclusions of this article are included in the article.

## Abbreviations

AZD7762: 1-(2-((*S*)-piperidin-3-ylcarbamoyl)-5-(3-fluorophenyl) thiophen-3-yl)urea
OS: osteosarcoma
Chk: checkpoint protein kinases
Cdc: cell division cycle
Cdk: cyclin-dependent kinases
IC50: half maximal inhibitory concentration
MTS: 3-(4,5-dimethylthiazol-2-yl)-5-(3-carboxymethoxyphenyl)-2-(4-sulfophenyl)-2*H*-tetrazolium
BSA: albumin from bovine serum
TUNEL: terminal-deoxynucleotidyl transferase mediated nick end labeling
EMEM: Eagle’s Minimal Essential medium
OD: optical density
PMSF: phenylmethanesulfonyl fluoride
Cis: cisplatin
