# Supplementary figures and images for: Checkpoint kinase inhibitor AZD7762 enhance cisplatin-induced apoptosis in osteosarcoma cells
Source: Cancer Cell Int. 2019 Jul 27;19:195. doi: 10.1186/s12935-019-0896-9 (PMC6660702; doi:10.1186/s12935-019-0896-9)

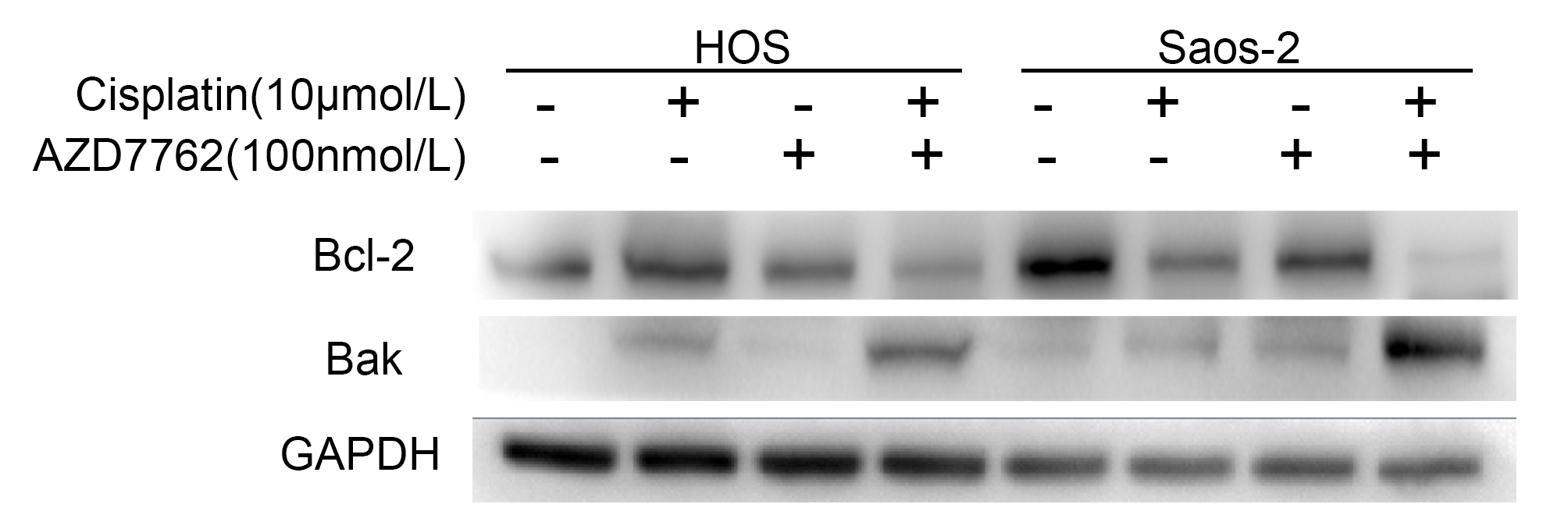

Supplement: Supplementary file 1 — Additional file 1. Human osteosarcoma cells HOS and Saos-2 were treated with PBS (control group), cisplatin (10 μmol/L), AZD7762 (100 nmol/L) and cisplatin + AZD7762 for 24 h The expression level of protein Bcl-2 and Bak were determined by western blot. [file 12935_2019_896_MOESM1_ESM.tif]

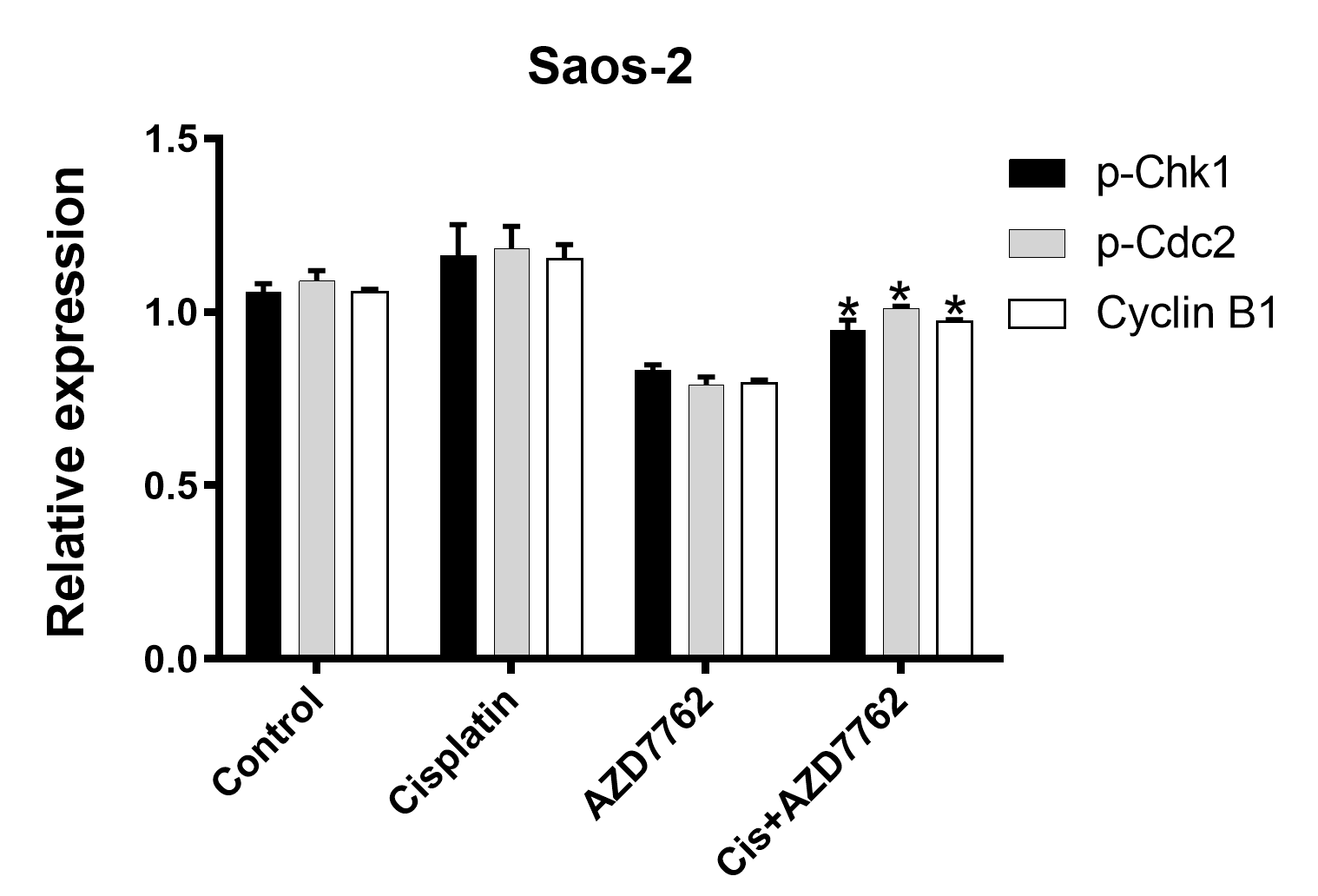

Supplement: Supplementary file 2 — Additional file 2. The relative expression of P-Cdc2, P-Chk1 and cyclin B1 in saos-2 cells were shown with bar graph. *p < 0.05 versus cisplatin treatment with the same concentration. [file 12935_2019_896_MOESM2_ESM.tif]

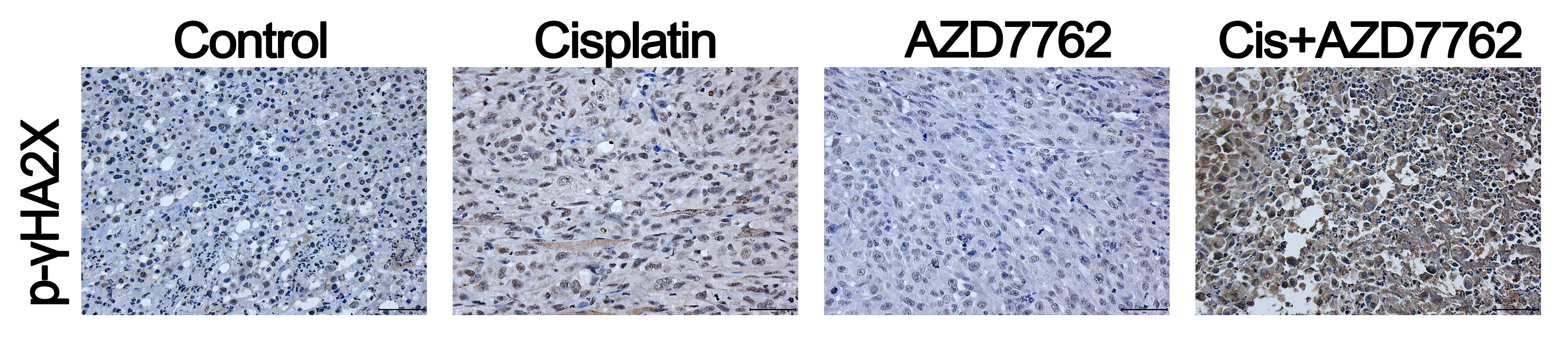

Supplement: Supplementary file 3 — Additional file 3. The high-resolution Immunohistochemistry images of p-γHA2X. [file 12935_2019_896_MOESM3_ESM.tif]
